# Suspended Slot Membrane Waveguide Based on Germanium-on-Silicon-on-Insulator at λ = 4.23 µm for CO_2_ Monitoring

**DOI:** 10.3390/mi15121434

**Published:** 2024-11-28

**Authors:** Muhammad A. Butt, Ryszard Piramidowicz

**Affiliations:** Institute of Microelectronics and Optoelectronics, Warsaw University of Technology, Koszykowa 75, 00-662 Warsaw, Poland

**Keywords:** suspended slot membrane waveguide, germanium-on-silicon-on-insulator, evanescent field absorption, Lambert–Beer law

## Abstract

In this work, we propose a novel suspended slot membrane waveguide (SSMW) utilizing a germanium-on-silicon-on-insulator (Ge-on-SOI) platform for carbon dioxide (CO_2_) gas-sensing applications. The design and analysis focus on the absorption line of CO_2_ in the mid-infrared region, specifically at a wavelength of 4.23 µm. The waveguide geometry has been precisely optimized to achieve a high evanescent field ratio (EFR) and minimize waveguide propagation losses. These optimizations significantly enhance the sensitivity of the waveguide, making it highly effective for evanescent field absorption-based gas sensing. Our optimized waveguide geometry demonstrates a notable EFR of 0.86, with a low propagation loss of just 1.07 dB/cm, and achieves a sensitivity as high as ~1.12 × 10^−4^ ppm^−1^ for SSMW lengths as short as 0.9 cm.

## 1. Introduction

**Figure 1:**
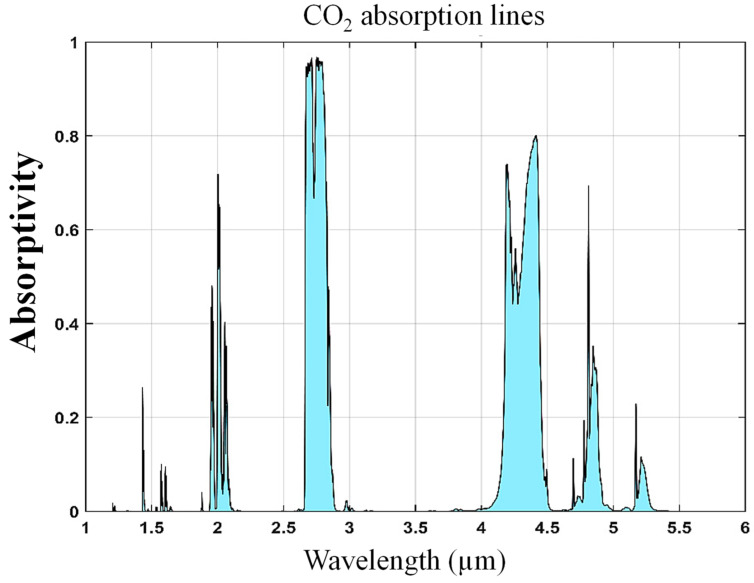
CO_2_ absorption bands in the SWIR–MWIR spectral range [12].

Monitoring CO_2_ gas is essential for environmental protection and sustainability [1,2]. It plays a crucial role in mitigating climate change by tracking greenhouse gas concentrations, which informs policies aimed at reducing global warming. Additionally, CO_2_ monitoring enhances air quality control, ensuring public health and safety by providing early warnings in areas with high emissions [3]. It also aids in ecosystem management by assessing the impact of CO_2_ on oceans and forests, guiding conservation efforts. Furthermore, monitoring CO_2_ helps improve industrial efficiency and supports the transition to renewable energy, leading to reduced emissions and a healthier environment [4]. CO_2_ becomes toxic when its concentration in the air rises, leading to harmful effects on the human body [5]. At high levels, CO_2_ displaces oxygen, reducing the amount available for breathing. This can cause symptoms like headaches, dizziness, confusion, and, in severe cases, respiratory distress or unconsciousness [6,7]. Prolonged exposure to elevated CO_2_ can lead to hypercapnia, a condition where excess CO_2_ in the bloodstream disrupts the body’s acid–base balance, impairing the function of vital organs and potentially leading to life-threatening consequences [8]. CO_2_ is detected primarily through its absorption of specific wavelengths in the mid-infrared (MIR) region of the electromagnetic (EM) spectrum. CO_2_ has strong absorption bands around 4.23 μm [9]. The absorption at 4.23 μm corresponds to the asymmetric stretching mode of the CO_2_ molecule, while the absorption around 15 μm is associated with its bending modes [10]. These absorption characteristics make CO_2_ detectable in atmospheric studies and industrial applications using infrared spectroscopy, where the attenuation of IR light at these wavelengths indicates the presence and concentration of CO_2_ [11]. Figure 1 illustrates distinct absorption bands within the SWIR to MWIR spectral range, highlighting key regions where specific wavelengths are absorbed.

Ge-SOI platforms offer a compelling solution for mid-IR sensing, characterized by several advantageous properties [13]. Ge’s broad transparency in the mid-IR range (2–18 µm) and high refractive index enable strong light confinement and enhanced light–matter interaction, critical for sensitive detection [14]. Integrating Ge with the mature SOI technology leverages existing silicon photonic fabrication processes, ensuring scalability and cost-effectiveness. This platform also exhibits good thermal stability and low propagation loss (α_prop_), maintaining signal integrity over longer distances [15]. Additionally, the high refractive index contrast between Ge and the silicon dioxide insulator layer boosts the performance of integrated photonic circuits [16]. These characteristics make Ge-SOI an ideal platform for developing advanced mid-IR sensors for applications in environmental monitoring, medical diagnostics, and industrial process control [16,17].

Photonic sensors play a pivotal role in gas sensing due to their high sensitivity, selectivity, and rapid response times [18,19]. Utilizing the interplay of light with gas molecules, these sensors can detect even trace amounts of gasses, making them irreplaceable for environmental surveillance, industrial safety, and healthcare applications [20]. Their ability to operate in real-time allows for continuous monitoring and the immediate detection of hazardous gasses, ensuring timely interventions [20]. Additionally, photonic sensors often benefit from compact designs and robustness, enabling deployment in various challenging environments. Their non-intrusive nature and potential for integration with other optical systems further enhance their versatility and effectiveness in gas-sensing applications [21,22].

Evanescent field absorption gas sensors hold significant importance because of their extraordinary sensitivity and selectivity in detecting trace gasses [23]. These sensors leverage the evanescent wave, a phenomenon occurring when light propagates through a waveguide and an exponentially decaying field extends beyond the waveguide surface into the surrounding medium [24]. When this evanescent field interacts with gas molecules near the surface, it can absorb specific wavelengths of light, causing detectable changes in the optical signal [25]. This method allows for real-time monitoring and the precise identification of various gasses at low concentrations, which is crucial for environmental monitoring, industrial process control, and healthcare diagnostic applications. The ability to detect minute changes in gas composition with high accuracy makes evanescent field absorption gas sensors invaluable in ensuring safety and regulatory compliance and enhancing the overall understanding of atmospheric and process gasses [26]. In contrast, polyhexamethylene biguanide (PHMB)-based sensors rely on chemical interactions, which can suffer from cross-sensitivity to other compounds and degradation over time [27,28,29].

Building on this phenomenon, a variety of photonic sensors have been developed to detect a broad spectrum of atmospheric gasses. These sensors leverage advanced optical techniques to attain high sensitivity and selectivity, permitting the accurate monitoring of gas concentrations in various environments. The versatility of these sensors makes them ideal for applications ranging from environmental monitoring to contributing significantly to our understanding and management of atmospheric conditions [24,30,31,32,33,34,35]. In this work, a numerical investigation of the low-loss and highly sensitive suspended slot membrane waveguide (SSMW) based on the Ge-SOI platform for the detection of CO_2_ gas at an operational wavelength of 4.23 µm is carried out.

## 2. Device Design and Numerical Model

**Figure 2:**
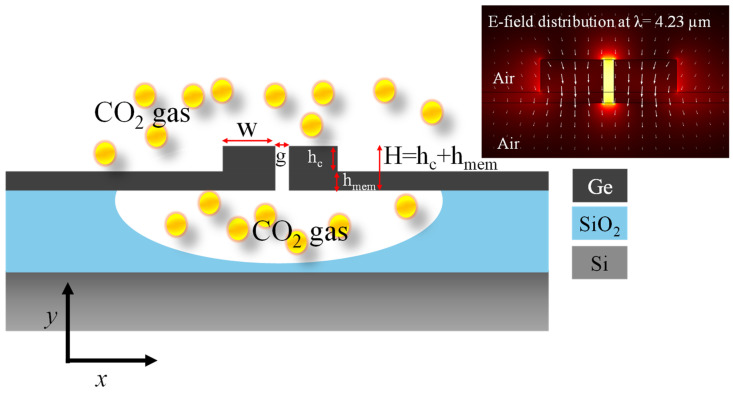
Schematic depiction of a Ge-on-SOI platform-based SSMW. Inset: normalized electric field distribution at λ = 4.23 µm.

SSMW is an innovative structure for gas-sensing applications, characterized by its enhanced sensitivity and interplay with the target gas [36,37]. The waveguide features a narrow slot denoted as “g” that runs along its length, suspended in air or another low-index material, which significantly increases the overlap between the optical mode and the surrounding environment [38]. This design maximizes evanescent field exposure, allowing maximum light to interact with the gas molecules, thereby improving detection sensitivity. The width and height of the rails are represented by W and h_core_, respectively. The rails are positioned on a suspended membrane of height (h_mem_), such that the total height (H) of the waveguide core is given by h_core_ + h_mem_. The graphical illustration of SSMW is shown in Figure 2.

The suspended nature of the membrane minimizes substrate losses and allows for high refractive index contrast, which enhances light confinement within the slot. In SSMW, the membrane is isolated from the substrate, making it more susceptible to mechanical deformations, such as bending or sagging, especially under external forces or thermal variations. An optimized membrane enhances the mechanical robustness, providing better resistance to these deformations, minimizing the α_prop_ and sustaining the EM-field’s confinement in the narrow slot of the waveguide. This combination of increased light–gas interaction, low losses, and integration capability makes SSMW a powerful tool for precise and reliable gas-sensing applications [39,40,41]. The set of parameters that are used in the optimization process of the waveguide are presented in Table 1.

The finite-element method (FEM) utilized in COMSOL Multiphysics 6.0 is vital for examining SSMW’s modal characteristics, as it enables the precise handling of complex geometries and material variations. COMSOL provides extensive material property data, including the refractive index, through its built-in material libraries [42]. These libraries encompass a broad selection of predefined materials, each with associated physical properties such as refractive index, thermal conductivity, density, specific heat, and electrical conductivity. For waveguide design, materials like Ge, Si, and SiO_2_ are selected directly from these libraries. FEM divides the waveguide structure into smaller elements, offering a high level of spatial detail for the EM-field’s distribution. This approach facilitates the precise calculation of modal characteristics, including effective refractive indices, mode profiles, and loss parameters [43,44]. To calculate the effective refractive index (n_eff_) of a waveguide, a simulation model of the structure is developed using COMSOL. The procedure begins with defining the geometry of the waveguide and assigning appropriate material properties to each region. COMSOL’s comprehensive material library offers predefined properties such as refractive index, thermal conductivity, and electrical conductivity, which can be assigned or customized as needed. The wave optic module then establishes the EM-wave equations that govern the system. Boundary conditions and initial parameters, including the incident light’s wavelength (typically 4.23 µm), are set. The model is subsequently meshed and solved to obtain the EM-field’s distribution. By analyzing these results, the real and imaginary components of n_eff_ are derived, typically through the propagation constant of the guided modes. This method enables the accurate characterization of the waveguide’s optical properties.

## 3. Evanescent Field Ratio and α_prop_ of SSMW

The evanescent field ratio (EFR), also referred to as the penetration depth ratio, measures the relative strength of the evanescent field in comparison to the total field within an optical waveguide. In the case of SSMW, it is well defined as the ratio of the intensity or power of the evanescent field in the upper cladding (UC)+ nanogap (g)+ under-etched (UE) part to the total field intensity (rails + substrate + UC + g + UE), which is as follows:

(1) $$EFR=\frac{\iint_{UC+g+UE}{\left|E(x,y)\right|}^{2}dxdy}{\iint_{rails+substrate+UC+g+UE}{\left|E(x,y)\right|}^{2}dxdy};$$

This parameter is vital for understanding the amount to which the evanescent field engages with its environment, directly influencing the sensitivity of different sensing applications. An increased EFR indicates a more significant interplay between the waveguide and its surroundings, making it ideal for sensing applications necessitating elevated sensitivity. Waveguide architectures are adjusted to accomplish the anticipated EFR by balancing factors such as waveguide geometry, material platform, α_prop_, and operating conditions, all aimed at enhancing sensing performance. Song et al. determined the EFR and α_prop_ for various waveguide structures [33]. For the strip waveguide, the EFR was 0.15 with a propagation loss of 0.023 dB/cm. The subwavelength grating waveguide exhibited an EFR of 0.38 and a propagation loss of 1.54 dB/cm. The slot waveguide had an EFR of 0.47 and a propagation loss of 0.98 dB/cm, while the subwavelength grating slot waveguide showed an EFR of 0.56 with a propagation loss of 2.96 dB/cm.

The α_prop_ in a waveguide can be quantitatively assessed by analyzing the imaginary part of the effective refractive index, denoted as Im(n_eff_). The n_eff_ is a complex quantity, where the imaginary component represents the waveguide’s attenuation characteristics. Specifically, Im(n_eff_) accounts for loss mechanisms such as absorption and scattering within the waveguide material. The α_prop_ measured in dB/cm is related to this imaginary part through the following relationship:

(2) $$\alpha (dB/cm)=\left(\frac{4\times \pi \times Im\left(n_{eff}\right)}{\lambda }\right)\times 4.343$$

where λ is the wavelength of the propagating light. This formula indicates that a higher Im(n_eff_) corresponds to greater attenuation per unit length.

## 4. Discussion

Enhanced evanescent fields play a crucial role in the realm of sensing technologies, offering significant advantages for various applications [45]. These fields, which occur at the interface between two media when light undergoes total internal reflection, are highly sensitive to changes in the local environment. The enhancement of the evanescent field typically achieved using specialized waveguides such as SSWG allows for the recognition of small variations in refractive index, molecular interactions, and the presence of specific analytes. This sensitivity makes enhanced evanescent fields particularly valuable for biochemical and medical sensing, where they can detect low concentrations of biomolecules, pathogens, or chemical substances with high precision [23].

**Figure 3:**
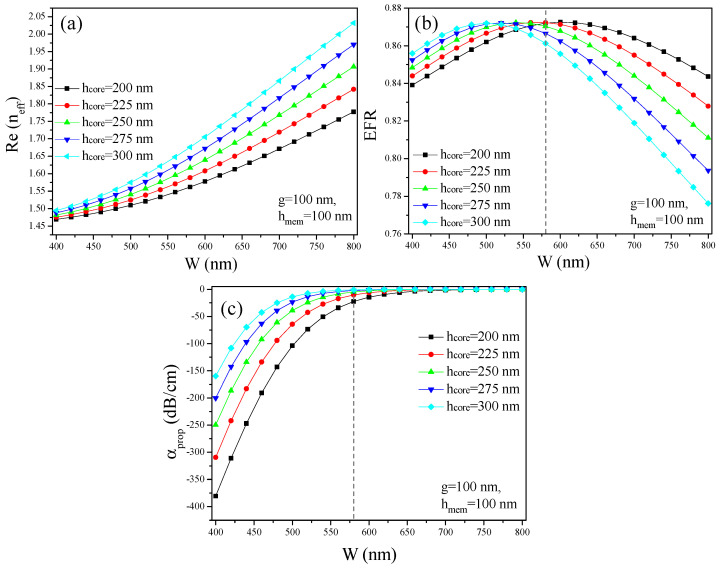
(**a**) Re(n_eff_) versus W, (**b**) EFR versus W, and (**c**) loss versus W.

In the first step, the waveguide geometry is optimized by keeping the nanoscale gap (g) and the membrane height (h_mem_) fixed at 100 nm. The Re(n_eff_), α_prop_, and EFR are plotted as shown in Figure 3a, 3b, and 3c, respectively. During this process, W is varied between 400 nm and 800 nm, whereas h_core_ is varied between 200 nm and 300 nm, with a step size of 25 nm. The Re(n_eff_) increases with an increase in both W and h_core_, indicating that the EM-field is highly confined in the nanogap of the slot waveguide. For instance, when h_core_ is kept constant at 300 nm, the Re(n_eff_) varies with changes in the W of the waveguide rail. Specifically, Re(n_eff_) is 1.49 for W = 400 nm, 1.70 for W = 600 nm, and 2.03 for W = 800 nm. This trend demonstrates that, as the W of the waveguide rail increases, the Re(n_eff_) also increases, further confirming the enhanced confinement of the EM-field within the nanogap and the rails of the slot waveguide. This relationship underscores the critical role of the waveguide width in tuning the optical properties of the device, which can be strategically utilized to achieve the desired performance characteristics in various photonic applications. Furthermore, the optimization of the waveguide dimensions aims to minimize α_prop_ while maximizing n_eff_, thereby improving the overall performance of the photonic device. 

However, high modal confinement leads to a lower EFR attributable to the reduction in the evanescent field around the waveguide, as most of the power is confined within the nanoslot and high-index rail. For instance, the EFR values are approximately 0.86, 0.855, and 0.78 for W of 400 nm, 600 nm, and 800 nm, respectively, while keeping the h_core_ fixed at 300 nm, as revealed in Figure 3b. Moreover, the α_prop_ of the waveguide is reciprocally related to the EFR value, as illustrated in Figure 3c. Specifically, the waveguide experiences a high α_prop_ of 159.6 dB/cm when the EFR is around 0.86 for W = 400 nm and h_core_ = 300 nm. This signifies that W = 400 nm and h_core_ = 300 nm are highly undesirable geometric parameters, which lead to a lossy waveguide. In contrast, the α_prop_ reduces to 0.001 dB/cm when W increases to 800 nm, with h_core_ remaining at 300 nm. This demonstrates that optimizing the waveguide dimensions can significantly reduce α_prop_, improving the overall performance of the waveguide.

**Figure 4:**
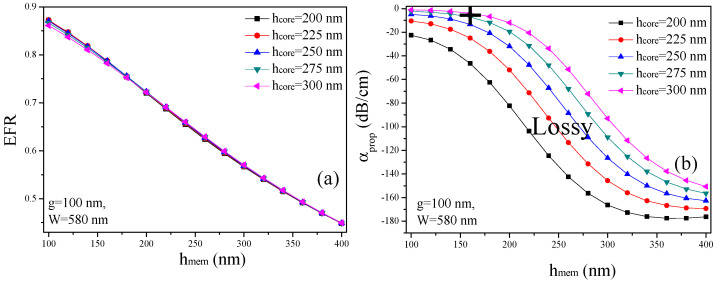
(**a**) EFR versus h_mem_ and h_core_, and (**b**) α_prop_ versus h_mem_ and h_core_. W and g are kept constant at 580 nm and 100 nm, respectively. The black cross in (**b**) represents the waveguide geometry (h_core_ = 300 nm and h_mem_ = 160 nm) where α_prop_ is significantly low.

The optimized thickness (h_mem_) of the suspended membrane is critical for achieving an ultimate waveguide geometry in SSMWs. This optimization ensures that the waveguide can effectively confine the light in the nanogap and guide it with minimal loss, enhancing the interplay between the optical field and the surrounding environment. An appropriately chosen h_mem_ balances mechanical stability with optical performance, leading to improved sensitivity and efficiency in applications such as sensing and signal processing. Consequently, the careful design and control of h_mem_ are fundamental to the overall performance and functionality of SSMWs. In this analysis, g and W are fixed at 100 nm and 580 nm, respectively, while the EFR is calculated for varying values of h_core_ and h_mem_, as shown in Figure 4a. Note that, W = 580 nm is selected because it provides low α_prop_ and high EFR, as estimated from Figure 3b,c.

It can be observed that the EFR declines as h_mem_ surges from 100 nm to 400 nm. This is because the EM-field is significantly confined within the membrane and the waveguide rails rather than the nanoslot. Additionally, h_core_ has a minimum effect on the EFR. However, α_prop_ is significantly high for h_core_ < 300 nm and h_mem_ > 160 nm, as illustrated in Figure 4b. For h_core_ = 200 nm, the α_prop_ ranges from 22.5 dB/cm to 46.3 dB/cm for h_mem_ between 100 nm and 160 nm. This shows that h_core_ = 200 nm is not appropriate if a low α_prop_ is required. For h_core_ = 300 nm, the α_prop_ ranges from 1.07 to 3.86 dB/cm for h_mem_ between 100 nm and 160 nm. However, h_mem_ < 100 nm is quite low for the mechanical stability of the waveguide, and h_mem_> 160 nm introduces significant waveguide loss. Therefore, considering mechanical stability and α_prop_, the SSMW must be designed in such a way that h_core_ must be maintained at 300 nm and h_mem_ can span between 100 nm and 160 nm.

**Figure 5:**
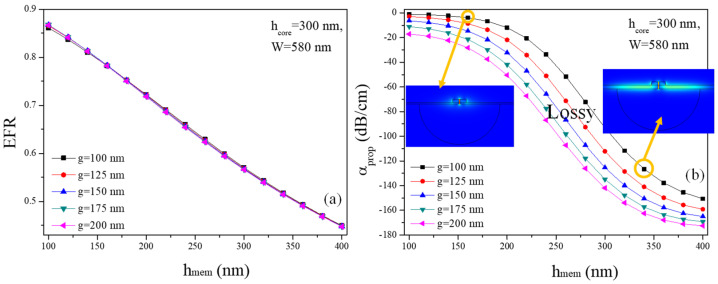
(**a**) EFR versus h_mem_ and g, and (**b**) α_prop_ versus h_mem_ and g. W and h_core_ are kept constant at 580 nm and 300 nm, respectively.

Ultimately, the EFR and α_prop_ of the SSMW are characterized as a function of varying h_mem_ and g, as depicted in Figure 5a and Figure 5b, respectively. W and h_core_ are held constant at 580 nm and 300 nm, respectively. The EFR decreases as h_mem_ increases from 100 nm to 400 nm, which can be attributed to the increasing confinement of the EM-field within the membrane and the rails. Interestingly, the parameter g exerts minimal influence on the EFR, offering flexibility in selecting g spanning 100–200 nm, as evidenced in Figure 5a. Conversely, optimizing the rail dimensions plays a crucial role in minimizing α_prop_ for h_mem_ values ranging between 100 nm and 400 nm. For instance, when g = 100nm, α_prop_ is measured at 1.07 dB/cm, 1.48 dB/cm, 2.31 dB/cm, and 3.86 dB/cm for h_mem_ of 100 nm, 120 nm, 140 nm, and 160 nm, respectively, as shown in Figure 5b. However, it is important to observe that EFR simultaneously decreases from 0.86 to 0.84, 0.81, and 0.78 as h_mem_ increases from 100 nm to 120 nm, 140 nm, and 160 nm. This indicates the intrinsic trade-off between maximizing EFR and minimizing α_prop_.

The EFR and α_prop_ for h_mem_ spanning a range of 100 nm to 200 nm are detailed in Table 2. This table provides a comprehensive overview of both desirable and undesirable values of h_mem_, offering insights into the optimal thickness for specific applications. The data highlight the balance between maximizing performance and minimizing potential drawbacks, enabling a more informed selection of h_mem_ values within the specified range. The SSMW structures with h_mem_ values of 100–160 nm are favorable due to their ability to achieve high EFR values (0.78–0.86) while maintaining low α_prop_. Conversely, SSMW structures with h_mem_ ≥ 100 nm are more prone to significant losses.

**Figure 6:**
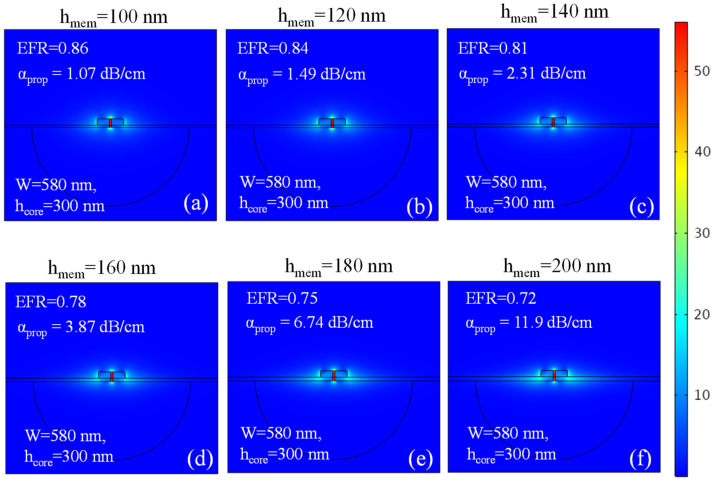
Normalized electric field profile in the SSMW of W = 580 nm, h_core_ = 300 nm, and g = 100 nm, whereas h_mem_ varies to (**a**) 100 nm, (**b**) 120 nm, (**c**) 140 nm, (**d**) 160 nm, (**e**) 180 nm, and (**f**) 200 nm.

Figure 6a–f depict the normalized electric field profile within the SSMW at a wavelength of λ = 4.23 µm for varying h_mem_. The other geometric parameters—namely, W, h_core_, and g—are held constant at 580 nm, 300 nm, and 100 nm, respectively. α_prop_ exhibits a linear dependence on h_mem_, indicating that minimizing h_mem_ is crucial for ensuring strict mode confinement within the nanoslot. If h_mem_ is greater than the cut-off waveguide layer thickness, then the mode extends into the membrane, which consequently leads to an elevated α_prop_. Gehl et al. successfully demonstrated a suspended membrane waveguide (SMW) with h_mem_ of 25 nm, 50 nm, and 75 nm, while maintaining the mechanical stability of the waveguide [40]. Therefore, we assume that, in the case of SSMW, h_mem_ spanning the range of 100 nm to 160 nm can offer good mechanical stability; however, the sensitivity of the waveguide varies based on h_mem_, as explained in the next section.

The fabrication of SMWs typically involves several precise microfabrication techniques to create a thin, free-standing layer that supports light propagation. The process begins with a substrate, onto which a thin film of the desired waveguide material is deposited. Photolithography is then used to pattern the waveguide structure, followed by reactive ion etching (RIE) to define the waveguide and remove excess material [46]. To create the suspended membrane, a sacrificial layer is etched away, often using a selective wet or dry etching process, leaving the waveguide structure suspended above the substrate. The holes/patterns are created during the photolithography and etching steps to allow the chemical etchant to access and remove the sacrificial layer beneath the waveguide. The size and placement of these patterns are carefully designed to balance the need for efficient etching with the mechanical and optical integrity of the suspended waveguide.

**Figure 7:**
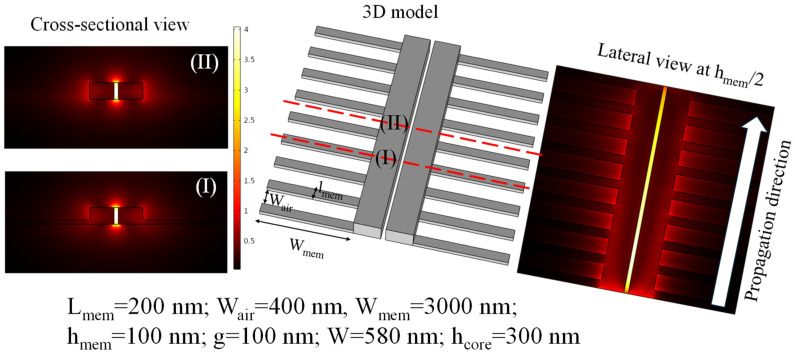
The three-dimensional model of the SSMW is in the (**middle**). The right side presents the lateral view of the normalized electric field profile in the waveguide at h_mem_/2. The cross-sectional view of the electric field profile is shown: (**top-left**) etched part of the membrane; and (**bottom-left**) unetched part of the membrane.

Qiao et al. designed an SMW utilizing a membrane width (l_mem_) of 200 nm and an air gap width (W_air_) of 400 nm [46]. In this design, l_mem_ refers to the width of the unetched membrane layer that forms the core of the waveguide, while W_air_ represents the width of the etched portion of the membrane, which creates a void or air gap beneath the suspended structure. This configuration is crucial for optimizing the optical confinement and minimizing losses in the waveguide. However, different values of l_mem_ and W_air_ can also be used based on the optimization process. The 3D model of the SSMW is designed as shown in Figure 7 (middle). On the right-hand side, a lateral view of the normalized electric field profile within the SSMW taken at h_mem_/2 is depicted. This view demonstrates that the mode is well confined within the nanogap and effectively propagates along the waveguide. Additionally, the bottom-left panel presents a cross-sectional view of the normalized electric field profile within the unetched portion of the membrane, as indicated by the red dotted line (I). Meanwhile, the top-left panel shows the normalized electric field profile within the etched region of the membrane, marked by the red solid line (II). These visualizations highlight the distinct field distributions in both the unetched and etched sections of the waveguide, emphasizing the role of each region in mode confinement and propagation.

## 5. CO_2_ Gas Sensing

CO_2_ gas has multiple absorption windows ranging from the NIR to the MIR region. At a concentration of 1 ppm under conditions of 296 K and 1 atm, the absorption coefficient (ε) of CO_2_ molecules can reach up to 3.778 × 10^−4^ ppm·cm^−1^ at a wavelength of 4.23 μm [47,48], which is four to five orders of magnitude higher than that in the vicinity of the 1.57 μm region. Lambert–Beer’s law, also known as Beer’s law, is a fundamental principle used in gas sensing to quantify the concentration of a gas based on the attenuation of light as it passes through a sample. According to this law, the amount of light absorbed by a gas is directly proportional to the concentration of the gas and the path length of the light through the sample. By measuring the decrease in light intensity at specific wavelengths, gas sensors can accurately determine the concentration of various gasses in a mixture. Based on Lambert–Beer’s law, the sensitivity of the SSMW is calculated using the following expression:

(3) $$Sensitivity=\frac{d(I/I_{o})}{d(c)}=-\eta \varepsilon L\exp\left(-\eta \varepsilon CL-\alpha_{prop}L\right)=-\eta \varepsilon LI_{n};$$

**Figure 8:**
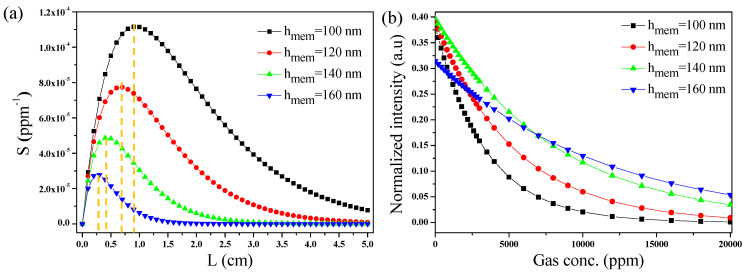
(**a**) Sensitivity of the SSMW versus L and (**b**) normalized intensity versus gas concentration.

A minus sign in the equation indicates the absorption phenomenon, while I_n_ = I/I_o_ represents the normalized intensity of the output light proportionate to the input light. Sensitivity is influenced by factors such as L and α_prop_, as well as η. These factors are critical for determining the sensitivity of the transducer. By differentiating both sides of Equation (3), it is determined that the sensitivity achieves its maximum value when L is set to 1/(ηεC + α_prop_). Figure 8a presents the sensitivity of an SSMW versus the L. From Table 2, it is evident that η decreases and α_prop_ increases as h_mem_ increases from 100 nm to 200 nm. This explains why sensitivity decreases as the waveguide length (L) increases, primarily due to the associated rise in total propagation losses. Additionally, h_mem_ significantly impacts the device’s sensitivity, with α_prop_ being higher for h_mem_ = 160 nm compared to h_mem_ = 100 nm. Consequently, it is crucial to optimize the waveguide’s geometric parameters to achieve maximum sensitivity. Fine-tuning these dimensions allows for a balance between minimizing propagation loss and enhancing light–matter interaction, leading to the most effective performance of the gas-sensing device. Table 3 summarizes the optimized lengths of SSMW for different values of h_mem_, where maximum sensitivity is obtained. This signifies that the sensitivity obtained by SSMW is significantly higher than the ones reported for subwavelength grating slots and slot waveguides that can reach up to 2.60 × 10^−5^ ppm^−1^ and 6.66 × 10^−5^ ppm^−1^ [33].

The normalized intensity (I_n_) is plotted against the different concentrations of CO_2_ gas using Equation (4):

(4) $$I_{n}=\exp\left(-\eta \varepsilon CL_{opt}-\alpha_{prop}L_{opt}\right);$$

The light propagating in the SSMW with h_mem_ of 100 nm decays more rapidly when exposed to CO_2_ gas compared to waveguide configurations with h_mem_ values of 120 nm, 140 nm, and 160 nm, as illustrated in Figure 8b. This is because η and L_opt_ obtained at h_mem_ = 100 nm are greater than other values of h_mem_, i.e., 0.86 and 0.9 cm, respectively (see Table 3). Moreover, with α_prop_ as low as 1.07 dB/cm, the waveguide exhibits a relatively longer interaction length, thereby enhancing its sensitivity and resulting in a faster decay of output power compared to the values reported for slot waveguides and subwavelength grating slot waveguides [33].

## 6. Conclusions

An SSMW based on Ge-on-SOI is highly advantageous for gas sensing in the MIR wavelength range. The MIR region is predominantly important for gas sensing because several gasses exhibit strong absorption features in this range. The high refractive index of Ge enables the tight confinement of light within the waveguide, enhancing light–matter interactions and enhancing the sensitivity of the sensor to trace gas concentrations. The suspended design minimizes optical losses by reducing the interaction with the substrate, thus improving the overall sensing performance. In this work, the SSMW is optimized at an operational wavelength of 4.23 µm, which links the absorption line of CO_2_ gas. Our optimized waveguide geometry exhibits an exceptional EFR of 0.86, combined with an impressively low propagation loss of just 1.07 dB/cm. This advanced design delivers outstanding sensitivity, reaching 1.12 × 10^−4^ ppm^−1^ for a waveguide length of only 0.9 cm, underscoring its potential for precision in MIR sensing applications.

Since the sensing mechanism is purely physical (based on optical absorption) and does not involve chemical binding or adsorption of CO_2_ molecules to the waveguide surface, the sensor offers excellent reusability. The interaction is non-invasive, and no permanent changes or degradation of the waveguide occur during operation. Therefore, the proposed structure can be reused indefinitely without performance loss, provided that the waveguide and optical setup are maintained properly. The insights gained from this research pave the way for the development of next-generation sensors capable of detecting trace gas concentrations with unparalleled accuracy, thus playing a vital role in addressing global environmental challenges.

## Figures and Tables

**Table 1:** Geometric parameters used in modeling.

Variable	Expression	Range (nm)
W	Width of the waveguide rail	400–800
g	Nanogap	100–200
h_mem_	Thickness of the membrane	100–400
h_core_	Thickness of the waveguide core	200–300
H	Total height (h_c_ + h_mem_)	300–700

**Table 2:** Characteristics of SSMW based on h_mem_. The results are extracted from Figure 5.

	g = 100 nm		
h_mem_ (nm)	EFR	α_prop_ (dB/cm)	Remarks
100	0.86	1.07	Highly desirable
120	0.83	1.49	Highly desirable
140	0.81	2.31	Highly desirable
160	0.78	3.86	Desirable
180	0.75	6.74	Least desirable
200	0.72	11.88	Lossy

**Table 3:** The sensitivity of the SSMW versus L. h_mem_ has a big influence on the waveguide sensitivity.

h_mem_ (nm)	L_opt_ (cm)	Sensitivity (ppm^−1^)
100	0.9	1.12 × 10^−4^
120	0.6	7.69 × 10^−5^
140	0.4	4.85 × 10^−5^
160	0.3	2.77 × 10^−5^

## Data Availability

Data will be available upon reasonable request.
